# ELDER ABUSE, ANXIETY, AND SOCIAL SUPPORT: DOES GENDER MATTER?

**DOI:** 10.1093/geroni/igad104.2870

**Published:** 2023-12-21

**Authors:** Sukyung Yoon

**Affiliations:** University of Wyoming, Laramie, Wyoming, United States

## Abstract

Elder abuse is a serious social justice violation and a public health concern that impacts victims’ mental and physical health worldwide. In the U.S., about 11.7% of older adults have reported elder abuse each year, but the rate is underestimated due to underreporting (Dong, 2015). To date, research on elder abuse has focused mostly on risk factors rather than protective factors (Storey, 2020). Some risk factors are beyond one’s control, such as physical and cognitive dysfunctions, but identifying both risk and protective factors will help healthcare professionals intervene more effectively (Yoon & Cummings, 2019). The research aims were to investigate 1) the impact of elder abuse on anxiety among community-dwelling older adults who experienced elder abuse, 2) the effects of social support on anxiety among older adults, and 3) the differences in the impact of elder abuse, anxiety, and social support between older men and women. This study used data from the Wisconsin Longitudinal Study (WLS), Wave 3. Structured equation modeling was used for the analysis. The independent variable was the experience of elder abuse, the dependent variable was anxiety, and the intervening variable was social support. The control variables were income, physical health, and marital status. The experience of elder abuse had a positive association with anxiety, however; social support had a negative association with anxiety. Multi-group analysis was conducted to investigate gender differences. Measurement invariance was examined by comparing unconstrained and fully constrained models. Both models were good fits. Gender differences were identified. Implications will be discussed.

